# Current Treatment Options for COVID-19 Associated Mucormycosis: Present Status and Future Perspectives

**DOI:** 10.3390/jcm11133620

**Published:** 2022-06-23

**Authors:** Yasasve Madhavan, Kadambari Vijay Sai, Dilip Kumar Shanmugam, Aashabharathi Manimaran, Karthigadevi Guruviah, Yugal Kishore Mohanta, Divyambika Catakapatri Venugopal, Tapan Kumar Mohanta, Nanaocha Sharma, Saravanan Muthupandian

**Affiliations:** 1 Department of Oral Medicine and Radiology, Faculty of Dental Sciences, Sri Ramachandra Institute of Higher Education and Research, Chennai 600116, India; yasasvem@gmail.com; 2 Centre for Biotechnology, Anna University, Chennai 600025, India; kadambarivijaysai@gmail.com; 3 Centre for Nanoscience and Nanotechnology, Sathyabama Institute of Science and Technology, Chennai 600119, India; shanmugamdilipkumar@gmail.com; 4 Department of Biotechnology, Sree Sastha Institute of Engineering and Technology (Affiliated to Anna University), Chennai 600123, India; bharathiaasha2@gmail.com; 5 Department of Biotechnology, Sri Venkateswara College of Engineering (Autonomous–Affiliated to Anna University), Sriperumbur 602117, India; karthigadevig@svce.ac.in; 6 Department of Applied Biology, School of Biological Sciences, University of Science and Technology Meghalaya (USTM), 9th Mile, Techno City, Ri-Bhoi, Baridua 793101, India; ykmohanta@gmail.com; 7 Natural and Medical Sciences Research Centre, University of Nizwa, Nizwa 616, Oman; 8 Institute of Bioresources and Sustainable Development (IBSD), Takyelpat, Imphal 795001, India; 9 AMR and Nanomedicine Laboratory, Department of Pharmacology, Saveetha Dental College, Saveetha Institute of Medical and Technical Sciences (SIMATS), Chennai 600077, India

**Keywords:** mucormycosis, COVID-19, fungal infection, risk factors, diagnosis, treatment

## Abstract

Mucormycosis has become increasingly associated with COVID-19, leading to the use of the term “COVID-19 associated mucormycosis (CAM)”. Treatment of CAM is challenging due to factors such as resistance to many antifungals and underlying co-morbidities. India is particularly at risk for this disease due to the large number of patients with COVID-19 carrying comorbidities that predispose them to the development of mucormycosis. Additionally, mucormycosis treatment is complicated due to the atypical symptoms and delayed presentation after the resolution of COVID-19. Since this disease is associated with increased morbidity and mortality, early identification and diagnosis are desirable to initiate a suitable combination of therapies and control the disease. At present, the first-line treatment involves Amphotericin B and surgical debridement. To overcome limitations associated with surgery (invasive, multiple procedures required) and amphotericin B (toxicity, extended duration and limited clinical success), additional therapies can be utilized as adjuncts or alternatives to reduce treatment duration and improve prognosis. This review discusses the challenges associated with treating CAM and the critical aspects for controlling this invasive fungal infection—early diagnosis and initiation of therapy, reversal of risk factors, and adoption of a multipronged treatment strategy. It also details the various therapeutic options (in vitro, in vivo and human case reports) that have been used for the treatment of CAM.

## 1. Introduction

Mucormycosis is a life-threatening invasive fungal infection (IFI), which, although once considered rare, has become increasingly prevalent in patients affected by SARS-CoV-2 [1]. The fungi responsible for mucormycosis belong to the order *Mucorales* and include genera such as *Rhizopus*, *Rhizomucor*, *Mucor*, *Lichtheimia*, *Cunninghamella* and *Saksenaea*. These fungi are commonly present in the environment. Although they are well recognized to cause opportunistic infections in immunocompromised patients, 19% of mucormycosis has been reported in immunocompetent patients [2,3]. A main reason behind recent mucormycosis infections is COVID-19 [4]. 

Depending on varying factors, mucormycosis infection is classified into five major types: rhino-orbital/rhino-cerebral/rhino-orbital cerebral mucormycosis (ROM/RCM/ROCM), pulmonary mucormycosis, cutaneous mucormycosis, disseminated mucormycosis and gastric mucormycosis. Various rare forms of mucormycosis are osteomyelitis, renal, peritonitis and cardiac [5]. This review focuses on the different types of mucormycosis, wherein ROCM/ROM/RCM mucormycosis commonly reported in COVID-19 is briefly discussed [6]. ROCM/ROM/RCM is caused by the colonization and spread through inhalation of fungal spores in the nasal pathways and surrounding regions [7]. Like RCM, pulmonary mucormycosis is also caused by the inhalation of fungal spores [8]. Cutaneous mucormycosis is an invasive form of infection which occurs through skin abrasions. It has been reported to be contracted through intravascular devices in a heart transplant patient affected by COVID-19 [9]. Intake of food contaminated by fungal spores causes gastrointestinal mucormycosis, which is usually rare in immunocompetent patients but has been reported in patients associated with COVID-19 infection [10,11]. This condition might also typically have a poor prognosis, especially if it disseminates to the heart, usually diagnosed during an autopsy [12]. Renal mucormycosis is commonly observed in COVID-19 patients with kidney transplants and is often associated with poor prognosis [13,14,15]. Mucormycosis peritonitis has been reported in patients undergoing dialysis [16]. Maxillary osteomyelitis associated with mucormycosis is quite common, resulting in pain, swelling, and bone exposure. Disseminated mucormycosis is a non-specific form that is widespread in the body due to the angio-invasive nature of the fungi [13]. 

Since early 2021, IFIs such as COVID-19-associated pulmonary aspergillosis (CAPA) and COVID-19-associated mucormycosis (CAM) have been increasingly found. CAPA, also called white fungus infection, primarily affects the lungs, and severely impacts the kidney, mouth, skin and brain. CAM, also called black fungus infection, primarily affects the nose and sinuses associated with COVID-19 but can also affect other areas depending on the sub-type [14]. Although more cases of CAPA were reported initially, the number of instances of CAM has progressively increased after the pandemic. It was reported by Pal et al., 2021 that the most significant number of mucormycosis infections were from India, which might occur due to the high prevalence of diabetes mellitus (DM) [1]. Mucormycosis is usually detected 13–18 days after development of COVID-19. However, many cases of CAM have been reported after the complete resolution of COVID-19 [15]. The high morbidity and mortality associated with CAM necessitate early treatment initiation [17]. This review focuses on the mechanisms of pathogenesis, risk factors, and various strategies used to treat CAM.

## 2. Mechanisms of Pathogenesis

Mucormycosis invasion occurs through glucose-regulated proteins (GRPs), which are molecular chaperones of the Hsp70 family (70 KDa Heat Shock Proteins) [18]. Although these are present in the endoplasmic reticulum (ER) under normal circumstances, ER stress conditions such as DKA, and the associated changes in tissue microenvironment (glucose, iron and ketone bodies), result in overexpression of GRPs in different compartments and the cell surfaces [8]. GRP78 is an essential receptor for adhesion and invasion of fungal hyphae and the resultant injury of endothelial cells [19,20]. The interaction with fungi is mediated by the fungal ligand spore-coating homolog protein (CotH) in *Rhizopus*, commonly CotH3 for ROCM. In pulmonary mucormycosis, invasion and infection are facilitated by fungal CotH7 with integrin-β1 (with heterodimer formation with integrin-α3) [20], which enables the superficial entry into the nasal epithelium. Further invasion involves attachment to the collagen IV and laminin in the extracellular matrix of the basement membrane of the endothelial cells [16]. Mucoricin, a ricin-like toxin produced by the fungi, may also aid this invasion and virulence [21,22]. Apart from adhesion, endocytosis is also responsible for causing damage to the host cells. Platelet-derived growth factor receptor (PDGFR) is involved in endocytosis and angioinvasion, which results in the dissemination of the infection and necrosis [23]. The mechanisms are discussed further along with risk factors to highlight the role of each element in causing disease.

## 3. Challenges in Control of Mucormycosis

The atypical clinical presentation of mucormycosis leads to increased disease spread, and hence early diagnosis is crucial and is the main target of current research. Direct examination, culture, and histopathology are the cornerstones of diagnosing mucormycosis, but they are time-consuming and lack sensitivity. Newer molecular diagnostic techniques, such as in situ hybridization and Polymerase Chain Reaction (PCR), offer an alternative that may lead to earlier diagnosis and prompt initiation of treatment [15]. Since mucormycosis is encountered during different phases of COVID-19, or even after recovery, high-risk patients should have regular follow ups [1].

Treatment of CAM is also complicated because early initiation of therapy is required to control the disease, but it should also be ensured that any empirical treatments for COVID-19 do not amplify the underlying co-morbidities, thus increasing the severity of the disease (e.g., steroid therapy causes immunosuppression, thus aggravating the disease) [24]. Additionally, mortality continues to be nearly 50% even after treatment [25]. Furthermore, since rural areas of India have limited access to health care facilities, this further adds to compromised treatment and increases mortality rates [15]. One of the most critical challenges is that a complete causal relationship between COVID-19 and mucormycosis is yet to be uncovered [26]. Hypotheses and possible associations between these two infections are discussed below.

## 4. Association of COVID-19 with Risk Factors of Mucormycosis and Their Role in Infection

The probability of acquiring mucormycosis is associated with various risk factors, of which the most important ones are DM (with or without ketoacidosis) and conditions causing immunocompromised status [27]. The primary risk factor affecting a population may also vary with geographical location. For example, in countries such as India, Iran and Mexico, the major pre-existing risk factor is DM, while primarily hematological malignancies are the main risk factor in Europe [5]. The predisposing condition may also determine the type of mucormycosis caused. Hematological malignancies and neutropenia are commonly associated with pulmonary mucormycosis, while DM is often related to rhinomaxillary and rhinocerebral disease [5,17,28]. Cutaneous mucormycosis is often associated with trauma or burns [5,9]. COVID-19, with or without immunosuppressive therapies, may act via various pathways to have a synergistic effect in creating an environment favorable for the development of CAM. Therefore, severe COVID-19 is considered a risk factor for mucormycosis. This section analyses CAM based on the link between COVID-19 and the various risk factors for mucormycosis. Additionally, the synergistic roles of these risk factors are explored.

### 4.1. Diabetes Mellitus and Diabetic Ketoacidosis

One of mucormycosis’s primary and most common risk factors is uncontrolled DM (especially with ketoacidosis). DM increases the severity of SARS-CoV-2 and the risk of mucormycosis [9], especially RCM. Mucormycosis seen in diabetic patients has clinical manifestations, including cranial nerve palsy, diplopia, mid-facial pain, proptosis, periorbital oedema, apex orbital syndrome, and palatal ulcers [7]. COVID-19 is responsible for an acute cortisol stress response, which may raise serum cortisol levels and hyperglycemia in both persons with and without DM [29]. 

Diabetes may be pre-existing or associated with COVID-19 infection (corticosteroid therapy for COVID-19 or other infectious diseases predisposes patients to mucormycosis) [27]. Diabetes or a hyperglycemic state is often associated with an inflammatory condition responsible for constant recruitment and activation of immune cells, which further exacerbates the inflammatory phenotype by increased secretion of proinflammatory cytokines. In these circumstances, antiviral immunity activation in response to SARS-CoV-2 infection also intensifies inflammation, which increases the chances of mucormycosis and other secondary infections [27]. DM promotes the growth and proliferation of fungal pathogens by affecting the immune system, affecting phagocytosis, chemotactic activity and transendothelial migration of neutrophils [30].

The virus affects angiotensin-converting enzyme 2 (ACE2) producing cells (including beta cells of the pancreas), leading to the decreased breakdown of angiotensin II. This causes insulin resistance and upregulation of the sodium and hydrogen exchanger (NHE). NHE can increase damage to the pancreas due to its role in insulin release [31]. NHE affects Na+ and Ca2+ transport, which leads to hypoxia [32]. This, along with COVID-19 associated cell lysis, leads to increased lactate levels, insulin resistance and endothelial damage. COVID-19 also causes lactic acidosis (accumulation of lactic acid), which further increases the activity of the NHE pump and increases the blood glucose level by gluconeogenesis. This also increases the serum iron concentration, which acts as a nutrition source for the growth of fungi [30].

Fungi of *Mucorales* are present generally in the environment [33]. They are opportunistic pathogens because normal human serum (at physiological pH range) can provide nutritional immunity against fungal invasion due to the iron-binding properties of transferrin and ferritin. This prevents fungi from getting access to iron for its functions [34]. However, COVID-19 may also cause diabetic ketoacidosis. Under the acidic conditions of diabetic ketoacidosis (DKA) (pH 4), this iron-binding ability reduces due to glycosylation of iron sequestering proteins, and so iron is no longer bound and utilized by the fungus for its disease pathogenesis [35]. 

Further, the favorable environment for fungal growth (high glucose levels, acidic conditions, ketone bodies such as β-hydroxy butyrate [BHB] and resultant free iron) created by DKA is responsible for increased expression of glucose-regulator protein 78 (GRP-78) on the surface of endothelium cells [8]. This interaction traps the inhaled spores in the nasal cavity, causing ROCM [20]. It is also involved in the entry of the SARS-CoV-2 and has been proposed as a potential drug target for targeting the virus [36,37]. As a result, invasion and injury of endothelial cells by *Rhizopus* is increased and tissue necrosis is observed [38]. DKA also causes immunosuppression by affecting T-lymphocyte induction, interferon-gamma and phagocytosis [8]. Additionally, administration of steroids in COVID 19 patients with pre-existing diabetes can affect phagocytosis by White Blood Cells and the destruction of pathogens by macrophages at various stages, making them more susceptible to *Mucorales* infections [38].

### 4.2. Immunosuppression

Prolonged administration of corticosteroid therapy or immunomodulatory drugs to patients with COVID-19 and pre-existing comorbidities can increase their risk of developing CAM. It was found that immunocompromised patients who crossed a threshold of 600 mg of prednisone (cumulative dose) or 2–7 g methyl prednisone (preceding month alone) are at higher risk of mucormycosis infection. In a study conducted by Patel et al. 2021, it was found that for the majority of the patients, the cumulative glucocorticoid dose administered vastly exceeded the recommended dosage. However, shorter courses of corticosteroid treatment of even 5–14 days have been found to predispose diabetic patients to mucormycosis [38,39]. Additionally, dexamethasone, a WHO-recommended corticosteroid treatment for severe or critically ill patients with COVID-19, has been associated with higher susceptibility to IFIs. These immunomodulatory and corticosteroid treatments and COVID-19 may affect phagocytosis and other immune responses [27]. Although steroid treatment in DM patients increases the risk of them developing CAM, the literature supports that patients without DM have also developed CAM after steroid use. Therefore, it is recommended that steroid therapy be avoided, especially in patients who exhibit mild COVID-19 [40].

It has been hypothesized that COVID-19-mediated ACE2 dysregulation creates a cascade that results in an environment suitable for fungal growth through its effects on the pancreas, lungs, colon, ileum, esophagus, cardiovascular and cardiovascular tissues [30]. ACE2 is ubiquitous on the lymphocyte surface and is likely involved in lymphocyte damage in COVID-19 infection [41]. COVID-19 is believed to cause immunosuppression due to lymphocyte damage by apoptosis due to the cytokine storm (which involves elevated levels of various proinflammatory cytokines such as several interleukins and TNF-α) and the resultant lymphoid tissue atrophy [30,42]. This cytokine storm also results in lactic acidosis, which has a detrimental effect on the proliferation of lymphocytes [43]. Together, these factors cause a reduction in lymphocytes (lymphocytopenia) [31]. SARS-CoV-2 infection lowers the levels of CD4 and CD8 T-cells. It also affects the responses of lymphocytes Th1 and Th2 (T helper type 1 and 2 cells) [44]. As a result, COVID-19 patients with acute respiratory distress syndrome (ARDS) exhibit immune system alteration and increased susceptibility to IFIs such as mucormycosis. Given the potential impact on the immune system, COVID-19 treatment with immunomodulatory drugs, such as IL-6 inhibitors, should be reserved for selected patients according to existing guidelines [40].

COVID-19 is also associated with a reduction in phagocytosis, thrombosis and endothelialitis [38]. Endothelial adhesion and penetration are crucial for mucormycosis entry and infection. The increased IL-6 levels in response to COVID-19 and acidosis also result in ferritin production, leading to intracellular iron accumulation, which damages the tissue. This tissue damage is responsible for releasing iron into the bloodstream, enabling fungus growth [45].

### 4.3. Nosocomial Sources

Mucormycosis may also be associated with nosocomial sources, especially during prolonged hospitalization [46]. Non-sterile equipment in hospitals is the main disseminator of infections among immunocompromised patients. Such equipment includes unsterilized/non-sterile bandages, nitroglycerin patches, ostomy bags, hospital linens, adhesive tape, wooden tongue depressors and even consumables such as probiotics, pre-packaged food and allopurinol tablets [5,47,48]. Medical apparatus and devices inserted into the body can allow direct access of fungal pathogens to infect the body. This includes intravascular devices such as IV catheters, lancets for insulin measurement, tubes inserted into the body, intubation, injections, and dental and surgical procedures [49]. A similar mode of infection is seen in intravenous drug abusers [38]. Prolonged ICU treatment can also increase the risk of mucormycosis, especially in patients under mechanical ventilation [50]. Environmental factors such as fungal pathogens in the air, water or surfaces in a hospital may also be responsible for hospital-associated mucormycosis. One such instance is the presence of oxygen humidifiers in hospitals which can spread potentially contaminated water, resulting in the significant spread of the disease [1]. Additionally, problematic plumbing and ventilation can augment the spread of infection among patients and lead to a community outbreak [5].

In the case of a heart transplant patient who did not demonstrate any of the usual risk factors associated with CAM, it was suggested that COVID-19 was responsible for lymphocytopenia and the resultant immunosuppression, which led to fungal infection [9]. The extent of respiratory pathology or pulmonary damage has been correlated with the nature of the risk of contracting CAM [51]. Intubation or mechanical (invasive) ventilation in the intensive care unit (ICU) for COVID-19 patients with ARDS for prolonged periods is a commonly observed risk factor for acquiring mucormycosis [52]. 

### 4.4. Other Factors

In general, treatment for COVID-19 with various antibiotics and immunosuppressive therapies such as monoclonal antibodies and steroids can cause dysbiosis of the human microbiome and damage epithelial linings, which aids the development of IFIs. One such treatment for COVID-19 is zinc, since it is known to have antiviral effects [53]. However, extensive use of zinc is significantly associated with occurrence of CAM since it promotes the growth of pathogenic fungi, without much benefit in treating COVID-19 [54,55]. Protracted treatment with antifungals for pre-existing fungal infections and a history of IFIs also increase the patient’s chances of being infected by *Mucorales* fungi [27]. Additionally, the renal tropism of the COVID-19 virus may also be responsible for kidney injury. Deferoxamine, administered to treat renal failure, is involved in iron sequestration by the *Mucorales* fungi, leading to mucormycosis [27]. In addition to all these aspects, in some cases, mucormycosis was observed even in COVID-19 patients without underlying predisposing factors, suggesting that the infection was responsible for creating a microenvironment favorable for the fungal population [56]. 

## 5. Diagnosis

Early diagnosis and intensive, multidisciplinary treatment and management of the disease are critical for a better prognosis. Intracranial extension was associated with a poor prognosis [57]. Hence early diagnosis is essential for better outcomes. Apart from clinical examination, imaging, histopathology, and culture are adjuncts for diagnosis.

### 5.1. Clinical Examination

Since early diagnosis is essential for a higher chance of patient survival, clinical examination plays a vital role in identifying clinical manifestations of patients with COVID-19 at moderate to increased risk of developing mucormycosis. This involves ocular examination as well as examination for sinus tenderness. The ocular examination involves testing for visual acuity, pupil and ocular motility, extraocular abnormalities, and examination of the fundus and biomicroscopy [52,57]. Abnormalities such as ophthalmoplegia, proptosis, blepharoptosis, affected visual acuity and perception of light, oedema and necrosis have been commonly observed in mucormycosis patients [58,59,60]. Intra oral examination should be performed to evaluate the presence of tooth mobility, swelling, tenderness and bone exposure [61].

### 5.2. Imaging

Imaging may not always be specific or diagnostic and the presentation may vary with the severity of mucormycosis. Computerized Tomography scan (CT), Magnetic Resonance Imaging (MRI, with/without contrast) and endoscopy are the standard imaging modalities used to assess the extent of involvement in mucormycosis. Staging is usually done based on sinus and cerebral involvement. Radiological imaging usually can be done by CT or MRI, with or without contrast. CT and MRI have been used to ascertain the extent of the fungal invasion and intracranial extension and, thus, the disease progression of mucormycosis. For this purpose, brain MRI is required as it helps ascertain brain, orbit and sinus involvement. MRI of orbits or paranasal sinuses may also be used to diagnose mucormycosis [62,63]. MRI has been found to detect the extent of the participation in mucormycosis with a higher degree of sensitivity when compared to CT. The most distinctive feature of mucormycosis visualized by an MRI is a peri-sinus invasion [28]. 

CT imaging may be performed for the paranasal sinus, nose, orbits, brain or chest for diagnosis. Bone destruction is generally observed using CT imaging of the paranasal sinus and brain. The presence of mucormycosis may be identified in CT at early stages using features such as a reverse halo often seen in the periphery of the lung. This might also be visualized as central necrosis and an air crescent sign [13,64]. Diagnostic features such as opacifications in the paranasal sinuses and orbits, optic nerve or mucosal thickening, fluid collection and inflammation can also be seen using CT.

Endoscopy can be performed alone or in combination with other procedures for diagnostic purposes, and may be rhinoscopy, sinonasal endoscopy or bronchoscopy for ROCM. Bronchoscopy detects tissue masses that obstruct the bronchus [47]. Further investigations are required to determine if this is due to fungi or a tumor. Endoscopy usually detects pus, blackish necrotic tissues, lesions and destroyed or damaged tissues. Alternatively, a minimally invasive procedure, called functional endoscopic sinus surgery, can be used. Esophago-gastroduodenoscopy can detect uncharacteristic necrotic ulcers (exudate), especially in COVID-19 patients, to diagnose GI mucormycosis [11].

### 5.3. Histopathology

Histopathology is the best approach for diagnosing mucormycosis due to its sensitivity and specificity [13]. Histopathological examination is conducted on samples from the palate, nasal samples, gastric ulcers, skin lesions and biopsy during endoscopy, and surgical debridement and post-operative samples. It usually confirms the presence and diagnosis of mucormycosis. Hematoxylin and eosin (H&E), Periodic acid-Schiff (PAS) and Gomori methenamine silver (GMS) are histological stains used for the identification of *Mucorales* structures [58]. Pauci-septate or aseptate, irregular, broad, filamentous hyphae branched at right angles and spores are typical features of mucormycosis under biopsy. Biopsy may also reveal necrosis, ulcers, granulation, inflammation, exudates, angioinvasion and vasculitis [11,47,58,60].

### 5.4. Culture

The microscopic examination of the exact fungi and the fungal hyphae can be done using culture. A nasal swab is usually used for a sample collection from suspected mucormycosis patients. This sample is viewed under a microscope by preparing a direct smear with 10% KOH to detect fungal colonies and hyaline mycelium [24,38]. However, mucormycosis may not always give rise to growth in culture, and may provide a false-negative result. Additionally, the layered appearance of the fungal ball may cause it to be misdiagnosed as allergic rhinosinusitis in low power microscopes, which can be avoided using high power microscopes [52]. Fluorescence brighteners can also be used to distinguish the colonies. Alternatively, samples such as tracheal aspirate, bronchial aspirate, bronchoalveolar lavage fluid (BALF), sputum, skin lesions and operative samples can also be collected and analyzed [4,65]. Fungi in these samples may be grown on Sabouraud Dextrose Agar (SDA) at 25–37 °C. The fungal structures can be visualized by staining using lactophenol cotton blue. Culturing on SDA can also be a confirmatory test [60].

Fungal colonies are usually detected based on morphological features such as color (cottony black, white or grey), but more specific tests such as DNA-sequencing can also be carried out. This may involve sequencing rRNA or 18S, 28S, internal transcribed spacer (ITS), and other barcode genes. MALDI-TOF spectrometry can also be used for confirmatory tests. Owing to fungal colonization, fungal DNA may be detected in various clinical samples such as tissue and serum. However, this approach requires further standardization [65].

Other non-invasive diagnostic techniques include quantitative multiplex polymerase chain reaction (qPCR) of blood serum targeting 18S rRNA of *Mucorales* fungi. qPCR-based detection designed by Million et al., 2006 was found to aid in early diagnosis by detecting *Mucorales* DNA at least three days before diagnosis of mucormycosis in over 90% of the study patients [66,67]. Commonly observed *Mucorales* genera such as *Mucor*, *Rhizopus*, *Lichtheimia*, and *Rhizomucor* have been detected using in-house assays. Since the use of these non-invasive methods for detection aid in early diagnosis and improved survival rate, qPCR result is also considered in addition to the reverse halo in the CT for diagnosis [13]. DNA can also be manually extracted and amplified using semi-nested PCR with primers specific to *Mucorales* and the resultant amplicon can be sequenced [29]. Additionally, MucorGenius^®^, developed by Pathonostics (Maastricht, The Netherlands), is an easy-to-use multiplex PCR assay for detecting *Cunninghamella* spp. in addition to the above clinically relevant *Mucorales* in BAL and serum [27]. Alternatively, other molecular methods such as Restriction fragment length polymorphism (RFLP) and melt curve analysis of PCR products enable earlier diagnosis with 70 to 100% sensitivity, making them valuable diagnostic tools [68].

Biomarkers such as *Mucorales* specific antigens have not been found in the blood serum of mucormycosis patients. So, unlike most IFIs, antigen tests such as the galactomannan test and detection of (1,3)-b-D-glucan (BDG) are not used for the detection of mucormycosis [29]. Moreover, *Mucorales* fungi are not detected in the cerebrospinal fluid culture of ROCM patients. However, T-cells such as CD4+ and CD8+ are seen explicitly in invasive mucormycosis and have been suggested as a possible non-invasive diagnostic test for mucormycosis. These T-cells may be detected using enzyme-linked immunospot (also known as ELISpot) [68]. Once the pathogen is identified, antimycotic susceptibility testing (using reference methods such as CLSI (Clinical and Laboratory Standards Institute) and EUCAST (European Committee for Antimicrobial Susceptibility Testing) or commercial tests such as Etest) is carried out so that the physician can determine the course of treatment [69].

## 6. Current Recommended Strategies for Treatment of CAM

CAM displays a high degree of angio-invasiveness. As a result, a multi-pronged approach is required to control the disease and prevent a recurrence. The treatment strategy for CAM is similar to that of mucormycosis. It primarily involves three aspects: addressing risk factors and co-morbidities, surgical debridement of infected tissue and administration of antifungals to control the spread of infection [4,70]. Adjunctive therapies may also be utilized depending on individual patient presentation and history. However, an early diagnosis is the most critical aspect of treatment.

### 6.1. Reversal of Risk Factors

Reversal of risk factors involves reversing hyperglycemic, immunosuppressed states and other risk factors that perpetuate mucormycosis in patients with COVID-19. The immunosuppressed condition may be changed by tapering or discontinuing immunosuppressants such as corticosteroids, antimetabolites, and calcineurin inhibitors. In the case of CAM-affected transplant patients, this might not be possible, and so the patient is treated with corticosteroid monotherapy and cessation of all other drugs [44]. Glucose levels must also be strictly controlled using insulin therapy and antidiabetic drugs, while ketoacidosis must be promptly treated [68]. Neutropenia management was found to have less severe implications in mucormycosis when compared to DM and corticosteroid therapy. These co-morbidities must be kept under control even after discharge to prevent recurrence [57].

### 6.2. Surgical Debridement

The surgical part of the treatment involves otorhinolaryngology, ophthalmology, neurosurgery, oral and maxillofacial surgery [5]. Hoenigl et al., 2021 demonstrated that surgical intervention and systemic antifungal therapy were associated with improved outcomes compared to antifungal therapy alone for patients with COVID-19 affected by rhino-orbital cerebral mucormycosis without central nervous system (CNS) involvement [27]. Due to the angio-invasive nature of mucormycosis, surgical debridement is an essential part of the treatment regime. It is usually performed using endoscopy or functional endoscopic sinus surgery. As for mucormycosis, sinus debridement must be performed repeatedly, intensively, and regularly to control CAM [7,65]. It should be widespread and completed at the earliest, removing all black, necrotic tissues for improved prognosis. Usually, surgical debridement is easier and more useful for ROCM and soft tissue infection than for pulmonary mucormycosis. It is not of much use for mucormycosis infections, which are disseminated in the blood or are found in inaccessible regions. For pulmonary mucormycosis, the thoracic cavity may be debrided, and in more critical cases, lung transplantation may be required [13]. For extreme, threatening cases, orbital exenteration is a last-resort technique for patient survival. This includes patients who did not respond well to the systemic antifungal medication and developed symptoms such as lack of light sensitivity, necrosis of the orbits and total ophthalmoplegia [4,57]. Following surgical debridement/orbital exenteration, the tissues are sent for histopathological and microbiological examinations to ensure that clear margins have been obtained. In the absence of clear margins, further debridement may be required [63]. Following surgical treatment, facial reconstruction or prosthetic rehabilitation might be necessary, especially for patients with orbital exenteration, to improve their quality of life [71]. As this surgery is associated with the spread of infectious aerosol particles, appropriate personal protective equipment (PPE) and precautions must be used by surgeons, while debriding CAM-infected tissue [72].

### 6.3. Systemic Antifungal Therapy

Since *Mucorales* are resistant to many antifungals, the current first-line therapy against mucormycosis involves polyenes such as intravenous liposomal Amphotericin B (LAmB) (polyene). In contrast, salvage therapy includes IV posaconazole and isavuconazole (triazoles). However, systemic antifungal therapy is considered an adjunct to surgical debridement [11]. Cytokines can also be administered along with antifungal drugs for improved antifungal effects [68].

**Figure 1:**
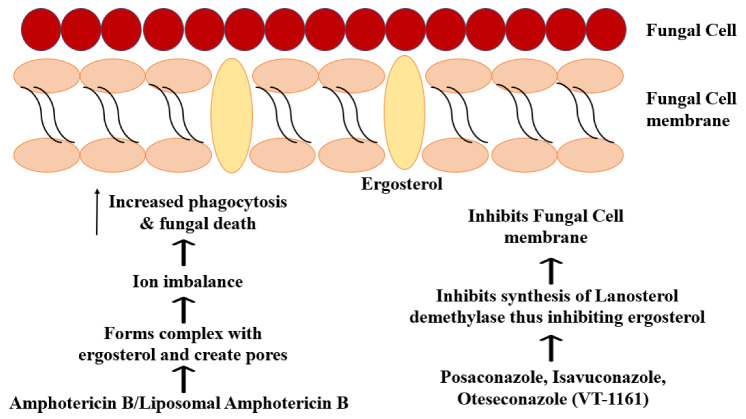
Drug action of Amphotericin B and azoles in the fungal cell membrane.

Amphotericin B deoxycholate and Amphotericin lipid complex (ABLC) have also been used. LAmB is preferred due to its reduced nephrotoxicity (especially at higher doses), improved CNS penetration and results in a murine model [13]. Amphotericin B deoxycholate is highly toxic, causing cholestasis and renal failure [47]. Consequently, if Amphotericin B is administered, monitoring kidney function is crucial. Amphotericin B acts on ergosterol, affecting the ion balance of cells, variations in membrane permeability due to oxidation and increased phagocytosis (Figure 1) [73]. Amphotericin B administered in cases of CAM varies from 3 mg/kg/day to 5 mg/kg/day or even 10 mg/kg/day in some cases, depending on the condition and co-morbidities of the patient [5,7,13,56,57,58]. Administration may be oral, intravenous or topical. Salehi et al., 2020 proposed the combination of LAmB, posaconazole and endoscopic surgical debridement (without craniotomy) as a treatment for ROCM patients who are not eligible for or willing to undertake extensive surgery [74]. Intranasal delivery of Amphotericin B (using nebulisation) in combination with systemic LAmB administration was favoured by Raj et al. 1998 [75]. Amphotericin B susceptibility also varies between different species of *Mucorales*. The duration of first-line treatment must be adjusted as per the co-morbidities and response of the patient, assessed by diagnostic tests. As amphotericin B is a fungistatic agent, the treatment duration is protracted compared to fungicidal agents. Polyenes such as LAmB have also been combined with echinocandins (which have low activity when used as monotherapy) such as caspofungin or micafungin and iron chelators such as deferasirox that control angioinvasion and pathogenesis and improve survival [13]. These combination therapies fall under second-line treatment options.

A double-blind placebo-controlled study by Spellberg et al., 2012 found that deferasirox was associated with higher mortality and lower success rate. Still, they could not draw generalized conclusions due to imbalances in the populations of deferasirox and placebo arms [76]. Amphotericin-B/LAmB/ABLC combinations have been tested with various drugs to treat mucormycosis with varying effectiveness. The combinations tested against mucormycosis and CAM are listed in Table 1 and Table 2. Posaconazole is active in vitro and in vivo (murine models) against various *Mucorales* fungi but demonstrated poor activity against *M. circinelloides*-infected mice [69]. It prevents fungal cell wall synthesis by inhibiting ergosterol biosynthesis through its action on CYP51, the fungal cytochrome P450 lanosterol 14-alpha-demethylase involved in ergosterol biosynthesis conversion lanosterol to ergosterol. This inhibition reduces ergosterol levels, thereby affecting the fungal cell membrane, causing the death of the fungus. Mutation of this gene can cause resistance [13,77]. It is used for salvage therapy and prophylaxis against Mucormycosis in patients with Graft-vs-host disease and high-risk factors [43]. It has also been used as part of the first-line treatment for some CAM patients, especially patients for whom amphotericin B cannot be used, or in cases where the infection has been controlled by initial Amphotericin B treatment. However, as Mucormycosis infections occur despite posaconazole prophylaxis, it is not the preferred drug for first-line treatment. It may be administered intravenously in the form of a delayed-release tablet or even as a syrup [65,78]. 

Isavuconazole, administered intravenously or orally, is an extended-spectrum antifungal, which is the reason for its use in the treatment of invasive mucormycosis [63]. It is used as a second-line drug for salvage therapy for CAM patients [4,63,65]. Due to the low hydrophilicity of isavuconazole, it is administered as a hydrophilic prodrug, isavuconazonium sulphate, which is converted to isavuconazole by esterase-mediated hydrolysis. As a result, unlike other azoles, it does not require cyclodextrin (likely to cause nephrotoxicity) to ensure drug solubility. So, it has a good safety profile in addition to being absorbed easily and having linear pharmacokinetics. It acts by inhibiting the synthesis of the fungal cell membrane. Like the other azoles, isavuconazole accomplishes this by inhibiting CYP51 of the CYP superfamily (cytochrome P450 monooxygenase). 

Itraconazole has also been limited activity and therapeutic effect against mucormycosis, acting primarily against *Saksenaea*, *Lichtheimia* and *Rhizomucor* [79,80]. Fluconazole and voriconazole are not used to treat mucormycosis due to lack of activity and low activity, respectively, with mucormycosis arising despite voriconazole treatment in some cases [81]. The effects achieved in these combinations for complete remission in CAM are listed in Table 1 and Table 2.

**Table 1:** Drug Combinations used against different types of mucormycosis.

Serial Number	Combination/Regimen	Type of Study	Type of Mucormycosis	Organism	Diagnostic Tests	Risk Factors (If Applicable)	Details of Combination/Regimen for Treatment of Mucormycosis (and Other Antifungals)	Other Concomitant Treatment (If Any)	Effect and/or Outcome	Addl Details	Reference
1	LAmB + CAS + SD	Case report	RCM	*Mucor*	CT, Clinical Diagnosis, Histopathology	1. Acute Myeloid Leukemia 2. Chemotherapy 3. Neutropenia	1. Liposomal Amphotericin B 2. Liposomal Ampotericin B + Caspofungin (24 days) 3. Surgical Debridement 4. Caspofungin (45 days)	1. Cytarabine 2. Idarubicin 3. Mitoxantrone 4. Broad-spectrum antibiotics 5. G-CSF 6. Potassium supplements	No infection after 3 months	Addition of Caspofungin was associated with improvement in patient’s conditions (LAmB monotherapy had no response)	[82]
2	(LAmB → ABLC) + CAS + SD	Case report	Oromandibular	*Rhizopus oryzae*	Clinical Suspicion, CT, Histopathology	1. Diabetes mellitus 2. Acute Myeloid Leukemia 3. Chemotherapy	1. AmB-deoxycholate 2. Fluconazole (stopped upon suspicion of mucormycosis) 3. Liposomal Amphotericin B + Caspofungin (56 days, maintained even after surgery) 4. Surgical Debridement 5. ABLC (5 Weeks)	1. Idarubicin 2. Cytarabine 3. Tobramycin 4. Colimycin 5. Morphine 6. Imipenem 7. Amikacin 8. Vancomycin	Alive, no recurrence at 6-year follow-up		[83]
3	LAmB + MCF + SD	Case report	ROM	*Rhizopus oryzae*	CT, Histopathology	1. Diabetes mellitus 2. Hemodialysis for chronic renal failure	1. Insulin therapy 2. Liposomal Amphotericin B 3. Surgical Debridement 4. Liposomal Amphotericin B + Micafungin (Oral) (2 + 4 weeks) 5. Amphotericin B (Sinus irrigation)	1. Meropenem	No recurrence seen in 1 year follow-up		[84]
4	HBO + LAmB + DEF + S	Case report	Hepatosplenic	*Candida zeylanoides* from blood cultures	Histopathology	1. Febrile neutropenia 2. Minimally differentiated AML 3. Chemotherapy	1. Voriconazole (9 days) 2. Voriconazole + Caspofungin 3. Liposomal Amphotericin B + Deferasirox 4. Hyperbaric Oxygen Therapy (60 sessions) + LAmB (21 days) + Deferasirox (Throughout)	1. Cefepime → Meropenem 2. Vancomycin 3. Consolidation therapy—high-dose cytarabine	CT unremarkable after first consolidation therapy		[85]
5	HBO + LAmB + PSZ + CAS + SD	Case report	ROM	*Rhizopus*	CT scan, Culture	1. Acute Lymphoid Leukemia (ALL) 2. Chemotherapy	1. Liposomal Amphotericin B 2. Sinus debridement 3. Liposomal Amphotericin B + Caspofungin + Posaconazole 4. Hyperbaric Oxygen Therapy (19 sessions) (Caspofungin stopped after 1 week of HBO, Amphotericin B continued for 2 months) 5. Discharged with oral posaconazole (4 months)	1. Ceftazidime 2. Vancomycin 3. Consolidation chemotherapy	Favourable		[86]
6	IFN-γ + NVB	Case report	Gastric		Histopathology	1. Immunosuppression	1. Liposomal Amphotericin B + Posaconazole 2. Gastrectomy 3. Splenectomy 4. Immunoadjuvant therapy 5. Nivolumab (1 dose)		Immunosuppression reversed. Patient discharged at 80 days		[87]
7	DAmB + LAmB + SD + VAC	Case report	Skin and Soft tissue	*Rhizopus*	Histopathology, Culture	1. Bilineal leukemia (ALL and AML) 2. Chemotherapy	1. Fluconazole (discontinued on diagnosis of mucormycosis) 2. Liposomal Amphotericin B (8 weeks) 3. Surgical Debridment 4. Vacuum-assisted closure (VAC) therapy 5. Deoxycholate amphotericin B (Topical) (3 weeks)	1. Chemotherapy for AML (cytosine arabinoside, daunorubicin, and etoposide) 2. Chemotherapy for ALL (cytosine arabinoside and L -asparaginase) 3. Trimethoprim-sulfamethoxazole 4. Gentamicin 5. Vancomycin 6. Salvage chemotherapy (vinorelbine, thiotepa, gemcitabine, topotecan and dexamethasone) 7. Alternative salvage chemotherapy (6-mercaptopurine, imatinib and methotrexate) 8. Palliative chemotherapy—vincristine	Mucormycosis controlled; no recurrence. Patient died of unrelated causes		[88]
8	LAmB (i.v.) + SD + AMB (N)	Case report	Sinonasal	*Absidia corymbisera * (Now *Lichtheimia corymbifera*)	Histopathology	1. Acute promyelocytic leukemia 2. Chemotherapy	1. Liposomal Amphotericin B (intravenous) 2. Amphotericin B (nebulisation)		Alive, no recurrence at 6-year follow-up		[75]
9	ABLC + (PSZ → ISZ) + CAS + SD	Case report	Disseminated	*Cunninghamella*	Clinical suspicion, Microscopic examination, Immunohistochemistry, PCR, Sanger sequencing, CT	1. Acute Lymphoid Leukemia 2. Chemotherapy 3. Neutropenia	1. Voriconazole (discontinued later) + Granulocyte colony-stimulating factor (G-CSF) 2. Amphotericin B Lipid Complex + Caspofungin 3. Posaconazole (3 days) 4. Isavuconazole (101 days, initially combination therapy, later monotherapy)	1. Cefepime 2. Vancomycin 3. Clarithromycin	Patient observed to be well at 10-month check		[89]
10	DAmB + MCF + PSZ	Case report	Disseminated	*Rhizopus*	Histopathology	1. Preterm birth 2. Mother underwent chemotherapy before delivery	1. Amphotericin B Deoxycholate + Caspofungin 2. Amphotericin B Deoxycholate + Caspofungin + Posaconazole 3. Micafungin discontinued subsequently (AMB—7 weeks; CAS—4 weeks, PSZ—3 weeks)	1. Ampicillin + Gentamicin 2. Vancomycin + Gentamicin 3. Ampicillin + Gentamicin + Metronidazole			[90]
11	AMB + CAS + SD	Case report	RCM	*Rhizopus arrhizus*	Histopathology, Molecular identification	1. Diabetes mellitus	1. Amphotericin B (60 days) 2. Amphotericin B + Caspofungin (4 weeks)	1. Targocid 2. Cefaxone 3. Flagyl	No recurrence in over 4 years	Caspofungin inclusion was associated with rapid improvement in symptoms	[91]
12	LAmB + PSZ + CAS + SD	Case report	Disseminated	*Absidia corymbisera * (Now *Lichtheimia corymbifera*)	Microscopic examination	1. Chemotherapy 2. Osteosarcoma 3. Brief neutropenia 4. Malnutrition	1. Surgical debridement—Multiple 2. Liposomal amphotericin B + Posaconazole 3. Liposomal amphotericin B + Posaconazole + Caspofungin (1 month) 4. Liposomal amphotericin B + Posaconazole (3 months)	1. High-dose methotrexate and etoposide-ifosfamide	Culture negative after triple combination therapy		[92]
13	(LAmB → PSZ) + S	Case report	Disseminated mixed invasive	*Rhizopus*	Histopathology	1. Pancytopenia	1. Fluconazole (discontinued eventually) 2. Liposomal Amphotericin B (discontinued on Day 100) 3. Surgical removal of fungal abcesses 4. Splenectomy 5. Nephrectomy (partial) 6. Lower lobe wedge resection (left) 7. Posaconazole (6 months, initiated on Day 100)	1. Immunosuppressant therapy (rabbit anti-thymocyte globulin, methylprednisolone, G-CSF) 2. Imipenem–cilastatin 3. Vancomycin 4. Hematopoietic Stem Cell Transplantation 5. Cyclophosphamide 6. Rabbit anti-thymocyte globulin 7. Cyclosporin 8. Methotrexate	No residual abscess seen at 30-month follow-up MRI		[93]
14	LAmB + PSZ + SD + S	Case report	Disseminated Cutaneous	*Rhizomucor pussilus*	Histopathology	1. Acute Lymphoblastic Leukemia 2. Neutropenia 3. Chemotherapy 4. Steroid Therapy	1. Surgical Debridement 2. Lung resection 3. Liposomal Amphotericin B + Posaconazole (12 weeks)	1. Cefoperazone-sulbactam 2. Amikacin 3. Induction chemotherapy	Complete remission		[94]
15	(LAmB + CAS + VOR) → (LAmB + PSZ + TER + SD + LAmB (N) + ABLC (i.pl))	Case report	Disseminated	*Cunninghamella bertholletiae*	Histopathology, PCR	1. Acute Lymphoblastic Leukemia 2. Pancytopenia	1. Liposomal Amphotericin B 2. Voriconazole (Discontinued subsequently) 3. Caspofungin (Discontinued subsequently) 4. Posaconazole + Terbinafine 5. Surgical Debridement 6. Liposomal Amphotericin B (Nebulisation) 7. Amphotericin B Lipid Complex (Intrapleural)	1. Broad spectrum antibiotics 2. Chemotherapy	No recurrence at 30 month follow up		[95]
	LAmB + TER + PSZ	Case report	Disseminated	*Cunninghamella bertholletiae*	PCR, Culture	1. Acute Lymphoblastic Leukemia 2. Allogenic Stem Cell Transplant 3. Steroid Therapy 4. Diabetes mellitus 5. Iron overload	1. Voriconazole (Discontinued later) 2. Liposomal Amphotericin B 3. Liposomal Amphotericin B + Terbinafine + Posaconazole	1. Methylprednisolone 2. Etanercept 3. Mycophenolate mofetil 4. granulocyte-monocyte colony-stimulating factor (GM-CSF) 5. Simvastatin 6. Deferasirox	Patient died 3 years later (cause not mentioned)		[95]
16	LAmB + PSZ	Case report	Disseminated	*Rhizopus microsporus*	Culture, Clinical suspicion,	1. AML	1. Voriconazole (Discontinued later) 2. Caspofungin (Discontinued later) 3. Liposomal Amphotericin B + Posaconazole (5 months) 4. Allogenic HSCT 5. Posaconazole 6. Surgery	1. Broad spectrum antibiotics 2. Antithymocyte globulin + Tacrolimus + Etanercept	No residual fungal lesions at 18 months		[96]
17	LAmB + PSZ + DEF	Case report	Hepatic	*Rhizomucor pusillus*	Microscopic examination, Histopathology	1. AML 2. Chemotherapy 3. Neutropenia 4. HSCT	1. Liposomal Amphotericin B 2. Liposomal Amphotericin B + Posaconazole 3. Surgical Debridement 4. Discharged with posaconazole 5. Deferasirox		Favourable		[97]
18	LAmB + CAS + SD	Case report	RCM	*Rhizopus oryzae*	Histopathology	1. Diabetes mellitus	1. Liposomal Amphotericin B 2. Liposomal Amphotericin B + Caspofungin 3. Liposomal Amphotericin B (Discharge, 2nd hospitalization)	1. Maxillectomy 2. Endoscopic decompression of orbita 3. Functional endoscopic sinus surgery 4. Meropenem 5. Ciprofloxacin	Recurrence due to patient non-compliance. Patient expired due to sepsis		[98]
19	DAmB + RIF	Case report		*Rhizopus oryzae*	Bronchoscopy, Culture	1. Diabetic Ketoacidosis	1. Rifampicin + Amphotericin B		Culture and histopathology negative after 8 weeks. Died of unrelated causes 3 years later.		[99]
20	AMB + (PSZ → AFG)	Case report	Hepatic	*Mucor* spp.	Histopathology, Immunochemical testing	1. AML 2. Neutropenia 3. Chemotherapy	1. Amphotericin B (10 days) 2. Amphotericin B + Posaconazole (2 months) 3. Amphotericin B + Anidulafungin	1. Chemotherapy-azacitadine 2. Moxifloxacin 3. Valacyclovir 4. Voriconazole 5. Levofloxacin 6. Metronidazole	Liver lesions improved. Patient expired due to complications		[100]
21	AMB/LAmB + CAS	Retrospective study	ROCM, ROM (Coexisting pulmonary, cutaneous)	*Rhizopus* spp., *Rhizomucor* spp.	CT, MRI	1. Diabetes mellitus 2. Neutropenia 3. Steroid therapy 4. Cancer 5. Transplant	1. Caspofungin + Polyene (ABLC/LAmB) 2. Surgical Debridement		1 Patient who received combination therapy expired within 30 days		[101]

AMB—Amphotericin B; PSZ—Posaconazole; AFG—Anidulafungin; RIF—Rifampin; TER—Terbinafine; CAS—Caspofungin; FLU—Fluconazole; ABLC—Amphotericin B Lipid Complex; LAmB—Liposomal Amphotericin B; MCF—Micafungin; DEF—Deferasirox; DAmB—Deoxycholate Amphotericin B; SD—Surgical Debridement; S—Surgery; IFN-γ—Interferon-γ; NVB—Nivolumab; VAC—Wound Vacuum Assisted Closure; G-CSF—Granulocyte-Colony Stimulating Factor; HSCT—Hematopoietic stem cell transplantation.

**Table 2:** Combinational drug therapy used in the treatment of CAM.

Serial Number	Combination/Regimen	Type of Study	Type of CAM	Organism	Diagnostic Tests	Risk Factors Other Than COVID-19 (If Applicable)	Details of Combination/Regimen for Treatment of Mucormycosis	Other concomitant Treatment (If Any)	Effect and/or Outcome	Reference
1	AMB + (ISZ → PSZ) + TCR + HBO + SD + Maxillectomy	Case report	Rhinosinusal	*Rhizopus oryzae*	Endoscopy, Culture, Palate Biopsy	1. Kidney Transplant 2. Immunosuppression 3. Prolonged history of isavuconazole use and IFIs 4. Diabetes mellitus (No DKA) 5. Steroid therapy	1. Treatment with Amphotericin B and azole (initially Isavuconazole, later posaconazole to avoid resistance) for 5 months 2. Surgical Debridement—7 times 3. Total Maxillectomy 4. Reduction of steroid (prednisone) dosage 5. Tacrolimus (Before diagnosis of CAM and during CAM treatment) 6. Hyperbaric Chamber Therapy	Azithromycin Ceftriaxone Dexamethasone Piperacillin/Tazobactam	No recurrence of infection after 5 months	[44]
2	Fasciotomy + SD + LAmB + ISZ	Case report	Musculoskeletal	*Lichtheimia ramosa*	Culture	1. Immunosuppression (Steroid Therapy—prednisone, mycophenolate and tacrolimus) 2. Kidney transplant (graft dysfunction)	1. Liposomal Amphotericin B + Isavuconazole (24 days) 2. Isavuconazole for 3 months 3. Surgical Debridement—3 times 4. Fasciotomy	1. Immunosuppressants (IS): prednisone, mycophenolate and TCR 2. Hydroxychloroquine 3. Azithromycin 4. Lopinavir/Ritonavir 5. Heparin 6. Tocilizumab (400 mg)	Favourable	[44]
3	FLU + AMB + SD	Case report	Sino-orbital	*Rhizopus oryzae*	Culture Histopathology MRI	None	1. Surgical Debridement—2 times 2. Fluconazole 3. Amphotericin B (injection and lavage) 4. Discharged with prescription for continuation of Amphotericin B and Fluconazole	1. Remdesivir 2. Methylprednisolone 3. Dexamethasone 4. Piptaz 5. Metronidazole 6. Tobramycin 7. Nepalact TDS 8. Monocef	Favourable at 2 month review	[59]
4	LAmB + PSZ + Sinus debridement without craniotomy	Case report	ROCM	Not Mentioned	MRI CT Culture of biopsy sample	1. B-cell lymphoma 2. Chemotherapy 3. Neutropenia	1. Liposomal Amphotericin B 2. Liposomal Amphotericin B + Posaconazole for 4 weeks 3. Surgical Debridement—Multiple	1. R-CHOP (rituximab, cyclophosphamide, doxorubicin, vincristine, and prednisone) 2. CODOX-M/IVAC (cyclophosphamide, vincristine, doxorubicin, high-dose methotrexate/ifosfamide, etoposide, and high-dose cytarabine) 3. Meropenem 4. Vancomycin	Patient discharged after 12 weeks. No recurrence for upto patient’s death, 3 months after discharge (unrelated to ROCM)	[74]
5	Amphotericin B + Azoles	Multicenter Epidemiologic Study	ROM, ROCM, Pulmonary, Renal, Disseminated, Others	*Aspergillus* and *Mucorales*	Microscopy Culture Histopathology	1. Steroid Therapy 2. Diabetes mellitus	Amphotericin B + Posaconazole (Concurrent or sequential)	1. Glucocorticoid drugs 2. Tocilizumab	The survival rates of sequential combination therapy were found to be better at 6 and 12 weeks compared to concurrent and single antifungal therapy	[39]
6	AMB + PSZ	Descriptive multicentre study (Cross-sectional)	Orbital	Not Mentioned	Not Mentioned	1. Diabetes mellitus 2. Steroid Therapy 3. Neutropenia	1. Amphotericin B 2. Posaconazole (2 weeks) 3. Orbital exenteration		Alive	[102]
	AMB + PSZ + SD		ROM			1. Diabetes mellitus	1. Amphotericin B 2. Posaconazole (2 weeks) 3. Surgical Debridement	Dexamethasone	Alive	
	AMB + PSZ + CAS + SD		ROM			1. Diabetes mellitus 2. Steroid Therapy	1. Amphotericin B 2. Posaconazole (2 weeks) 3. Caspofungin (2 weeks) 4. Surgical Debridement	Dexamethasone	Alive	
	AMB + CAS + SD		ROM			1. Diabetes mellitus	1. Amphotericin B 2. Caspofungin (2 weeks) 3. Surgical Debridement		Alive	
	AMB + PSZ + SD		Sino-orbital			1. Diabetes mellitus 2. Steroid Therapy	1. Amphotericin B 2. Posaconazole (2 weeks) 3. Surgical Debridement	Dexamethasone	Alive	
	AMB + CAS + SD		Sinonasal			1. Acute Myeloid Leukemia 2. Chemotherapy 3. Neutropenia	1. Amphotericin B 2. Caspofungin (2 weeks) 3. Surgical Debridement	Dexamethasone	Alive	
7	AMB + Azoles	Review (Statistical Analysis)	ROM, ROCM, Pulmonary, Cutaneous, Gastrointestinal, Disseminated, Others	*Rhizopus arrhizus*, *Rhizopus microsporus*, *Rhizopus* spp., *Lichtheimia* spp. And *Mucor* spp.	-	1. Glucocorticoid usage 2. Diabetes mellitus 3. Solid Organ Transplant 4. Immunosuppressive therapies	Amphotericin B + Azole (Isavuconazole or Posaconazole) (Sequential) Details of surgical debridement in combination with antifungal treatment not provided.	Details for individual cases unknown	Details for individual cases unknown	[103]
8	LAmB + VRZ + PSZ + SD	Retrospective Interventional study	ROCM	Not Mentioned	Histopathology, Imaging	1. Diabetes mellitus 2. Steroid Therapy	1. liposomal Amphotericin-B+ Voriconazole 2. Posaconazole 3. Orbital exenteration 4. Surgical Debridement	cefoperazone + sulbactam		[25]
	LAmB + PSZ + SD		ROCM		Histopathology, Culture	1.Diabetes mellitus 2. Steroid Therapy	1. Liposomal Amphotericin-B 2. Posaconazole 3. Surgical Debridement	1. Methylprednisolone 2. Prednisolone		
	LAmB + PSZ + SD		ROCM		Diagnosed as possible *Mucor* based on clinical evidence and imaging	1.Diabetes mellitus 2. Steroid Therapy	1. Liposomal Amphotericin-B 2. Posaconazole 3.Surgical Debridement	1.Dexamethasone 2.Prednisolone 3. Gabapentin		
	LAmB + PSZ + SD		ROCM		Histopathology, Culture	1.Diabetes mellitus 2.Steroid Therapy 3.Existing Antifungal Therapy	1. Liposomal Amphotericin B 2.Posaconazole 3.Surgical Debridement	1. Prednisolone		
	LAmB + PSZ + SD		ROCM		Histopathology, Culture	1.Diabetes mellitus 2.Steroid Therapy 3.Existing Antifungal Therapy	1. Liposomal Amphotericin B 2. Posaconazole 3. Orbital enteration 4. Surgical Debridement	1. Dexamethasone		
9	AMB + PSZ	Case report	ROM	Not Mentioned	Histopathology	1. Diabetes mellitus	1. Insulin injections to control hyperglycemia 2.Surgical Debridement 3.Amphotericin B 4. Posaconazole	1. Remdesivir 2. Levofloxacin 3. Dexamethasone 4. Vancomycin 5. Piperacillin-Tazobactam	Patient alive and stable at 2-month and 7-month follow up check	[58]
10	LAmB + CAS + PSZ	Case report	ROM	*Rhizopus* spp.	Histopathology CT Culture	1. Hyperglycemia	1. Liposomal Amphotericin B (4 days) 2. (Liposomal Amphotericin B → Posaconazole) + Caspofungin 3. Glucose Management 4. Surgical Debridement	1. Remdesivir 2. Vancomycin 3. Cefepime 4. Dexamethasone	Patient died due to COVID-19 associated ARDS	[28]
11	AMB + ISZ + MCF	Case report	ROCM		Mucormycosis suspicion based on MRI	1. Diabetes mellitus 2. Diabetic Ketoacidosis 3. Steroid Therapy	1. Amphotericin B 2. Isavuconazole 3. Micafungin	1. Remdesivir	Patient expired on Day 4 due to poor prognosis and rapid decline	[104]
	AMB + ISZ	Case report	ROCM	*Rhizopus*	CT Culture	1. Diabetic Ketoacidosis	1. Amphotericin B (3 weeks) 2. Amphotericin B + Isavuconazole (10 days)	1. Remdesivir	Patient expired	

## 7. Successful Drugs and Combinational Therapies against CAM

### 7.1. Hyperbaric Therapy

In some patients, hyperbaric oxygen therapy (HBOT) or hyperbaric chamber therapy is used as adjunctive therapy to other conventional therapies to improve survival rates. The humidifiers for oxygen therapy must use sterile distilled water. HBOT involves the patient’s exposure to 100% oxygen at pressures above one atmosphere (usually 2–2.5 atmospheres) for multiple treatments. This increases the oxygen transport capacity of the blood by increasing the alveolar partial pressure of oxygen, thereby causing revascularization and tissue oxygenation, thus reversing hypoxia. Theoretically, this could increase the oxygen concentration to a fungicidal level. However, hyperbaric oxygen is usually found to be fungistatic [105]. On the other hand, this is also frequently associated with oxygen toxicity due to free radical generation [106]. It also corrects lactic acidosis, which is a risk factor for mucormycosis, and, as a result, increases the activity of Amphotericin B. Furthermore, it also acts by boosting the immune response and reduces the area to be debrided, and hence is recommended to be used along with surgical debridement [107]. It is recommended for diabetic patients [108]. HBOT was part of a successful treatment regimen with antifungal treatment and surgical debridement to control CAM in a kidney transplant patient [44].

### 7.2. Immunosuppressants Used for Transplant Patients 

**Figure 2:**
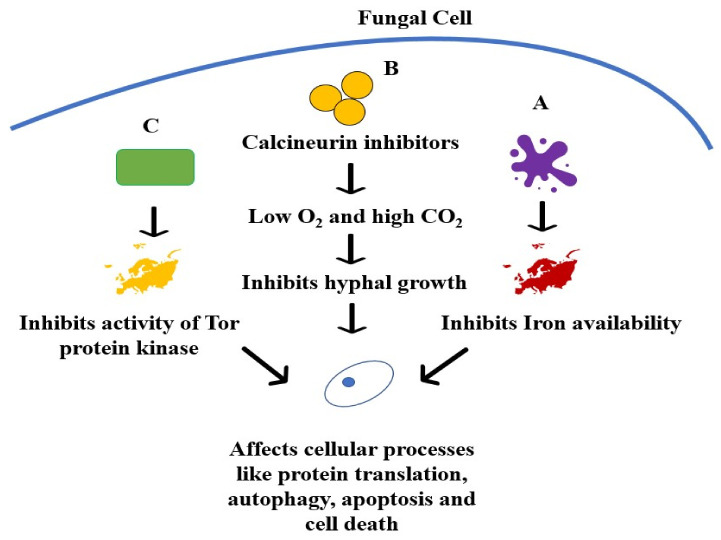
Drug action mechanism of (**A**) Deferasirox, (**B**) Calcineurin Inhibitors and (**C**) Rapamycin on the fungal cell.

These includes drugs such as calcineurin inhibitors (CNIs) and CNI alternatives such as sirolimus [109]. CNIs act against a conserved virulence factor, calcineurin, which is responsible for the hyphal growth of fungi. Calcineurin is central to virulence, morphogenesis and physiological processes. It is a serine/threonine phosphatase, which depends on a calcium-bound calmodulin binding to it for activation of phosphatase activity. Calcineurin inhibitors, which include drugs such as tacrolimus, act by reducing the virulence of mucormycosis, shifting from hyphal growth to yeast growth (lower virulence). CNI resistance occurs due to mutations in fkbA gene (which encodes for FKBP12, which binds to FK506 (sirolimus)), mutations in its binding sites (calcineurin catalytic A subunit or regulatory B subunit (cnbR)), and a mutation in both cnbR and bycA, which codes for an amino acid permease that regulates PKA activation. (Figure 2B) [110]. An epigenetic mechanism can induce transient or unstable resistance by RNA interference (RNAi) [111]. However, they primarily are used in combination studies as they increase the activity of other antifungals and demonstrate lower activity on their own. However, organ transplant patients treated with CNI as immunosuppressants showed reduced susceptibility to mucormycosis than those who did not receive CNI treatment. These combination studies have shown promising in vitro and in vivo results, but human trials are required [112].

Tacrolimus is a CNI used for transplant patients affected with Mucormycosis or CAM [44]. A study by Lewis et al., 2013 in mice showed that tacrolimus monotherapy prolonged survival while combination therapy was associated with close to complete resolution of lesions and symptoms [113]. Synergistic interactions were also observed in vitro at permissible human plasma concentrations. Notably, Tacrolimus was also a significant protective effect against mucormycosis in solid organ transplant patients [114].

Rapamycin (sirolimus) is an immunosuppressant drug that demonstrated in vitro and in vivo activity against *M. circinelloides* with improved survival rates (*Galleria mellonella* model) in a study conducted by Bastidas et al. 2012 [115]. They identified the drug targets as *M. circinelloides* homologs of FKBP12 (FK506-binding protein) and Tor (Target of Rapamycin) proteins. FKBP12 was critical for the inhibition of Tor (Figure 2C). FKBP12-Rapamycin inhibits Tor, which is involved in several cellular pathways dependent on nutrients. As a result, Tor inhibition causes nutrient starvation responses in the cell, leading to cell cycle arrest and autophagy. Although immunosuppressive therapies are usually tapered during mucormycosis patients, they are generally required for transplant patients. Consequently, rapamycin immunosuppressive therapy might help control mucormycosis in such patients. They suggested that the antifungal effects of rapamycin could be exploited with reduced or no immunosuppressive effects through combination therapy, modified delivery strategies such as lipid formulations, local delivery, topical applications or the use of non-immunosuppressive analogues of rapamycin [116,117].

### 7.3. Iron and Zinc Chelators

Iron is critical for the survival of *Mucorales* fungi. Consequently, sequestration of iron can be a strategy used to treat mucormycosis. Deferasirox is an iron chelator administered orally and may be fungistatic or fungicidal. It acts by affecting the iron availability to the pathogen, generating an iron-starvation response which terminates in metacaspase dependent apoptosis and cell death (Figure 2A). It was observed to have good activity in vitro and mouse models, increasing the survival period of mice. It demonstrated an activity comparable to that of LAmB in DKA mice and combination therapy demonstrated a longer survival time, but it did not lower the fungal burden consistently [59].

Additionally, Ibrahim et al., 2007 demonstrated that deferasirox showed higher activity against diabetic mice than in eutropenic mice, and that the activity was time-dependent rather than concentration-dependent [47], although the same combination was associated with higher mortality in clinical trials. The results might have been affected due to the small sample size (20 patients) and confounding factors such as variations in previous antifungal treatment and pre-existing conditions.

Zinc is a promoter of fungal growth, as demonstrated in an in vitro study of *Rhizopus arrhizus* strains isolated from CAM patients. This is due to its role in reducing the economic coefficient of the organism and facilitating the growth promoting activities of other micronutrients. However, the role of zinc in growth varies from strain to strain [55]. A study by Leonardelli recommended a combination of posaconazole with clioquinol, a zinc chelator, as it was found to be synergistic, especially against *Rhizopus microsporus.* Other combinations were also found to have synergistic activity, but varied from strain to strain [118]. 

### 7.4. Echinocandins

Echinocandins are combined with Amphotericin B to treat mucormycosis for synergistic effects. They inhibit cell wall synthesis in fungi by affecting the synthesis of BDG. The nature of the synergy remains unknown. These synergistic effects are observed with echinocandins such as caspofungin, micafungin and anidulafungin. They are used for the treatment of ROCM [119].

## 8. New or Repurposed Drugs

### 8.1. Drugs Used in Monotherapies

#### 8.1.1. VT-1161

VT-1161 is an investigational drug active in vitro against *Mucorales* species such as *R. oryzae*, *R. arrhizus*, *Lichtheimia* and *Cunninghamella*. It is a metalloenzyme inhibitor targeting the fungal CYP51 (such as isavuconazole), thus affecting cell membrane synthesis (Figure 1). VT1161 treatment performed favourably compared to posaconazole and LAmB, while prophylaxis by VT1161 was favourable compared to Posaconazole [120]. However, it was observed to have higher MICs than these existing therapies. VT1161 treatment and prophylaxis were also associated with increased survival and fungal burden reduction in neutropenic mice [121]. VT1161 has lower toxicity and better pharmacokinetics when compared to existing therapies such as azoles and polyenes. It also causes fewer off-target effects as it is selective to fungal CYP51 rather than CYP450 in humans. Further studies are required to evaluate the impact of this drug in experimental and therapeutic models.

#### 8.1.2. Manogepix

**Figure 3:**
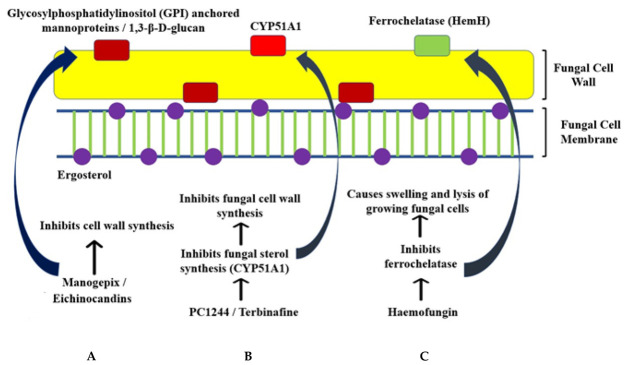
(**A**) Manogepix-mediated inhibition of mannoproteins. (**B**) PC1244-mediated inhibition CYP51A1. (**C**) Haemofungin-mediated inhibition of ferrochelatase.

Manogepix is a broad-spectrum antifungal agent that inhibits the conserved fungal protein Gwt1, affecting the trafficking and anchorage of mannoproteins to the cell membrane and outer cell wall. PIGW, the nearest ortholog in mammals, is not affected by Manogepix. Since mannoproteins are essential for fungi’s structural integrity and pathogenicity, Manogepix-mediated inhibition of mannoproteins can have various physiological and pleiotropic effects on growth and virulence (Figure 3A). It is more effective and has lower MICs and MECs (Minimum Inhibitory Concentration and Minimum Effective Concentration, respectively) for treatment of *Candida* and *Aspergillus*, and it usually exhibits higher MECs with *Mucorales.* However, it was demonstrated to be effective in two murine models of mucormycosis with low MECs, suggesting that using it for clinical treatment exists and must be explored further [122].

#### 8.1.3. Fosmanogepix (APX001)

Fosmanogepix is the prodrug form of Manogepix. Systemic phosphatases convert it to the active form of the drug, Manogepix. This pro-drug form is required due to the low solubility of Manogepix in water, making a delivery in an intravenous state complex [123]. It is now a first-in-class drug for the treatment of invasive mucormycosis. It demonstrated good activity, increase in survival and good tissue clearance in mouse models of invasive pulmonary mucormycosis. Fosmanogepix activity was comparable to isavuconazole [124] and found to have good pharmacokinetic properties, high bioavailability, widespread tissue distribution, and suitability for once-daily dosing in both oral and intravenous administration. It also has favourable interactions with other drugs and has no food effect. Consequently, it is currently in Phase 2 of clinical trials to treat infections caused by *Candida*, *Aspergillus* and rare moulds [125].

#### 8.1.4. Haemofungin

Haemofungin is an antifungal compound identified to affect cell wall synthesis leading to swelling and death. It targets HemH/ferrochelatase, thus preventing the final step of haem biosynthesis, leading to the accumulation of toxic intermediates, which also cause death (Figure 3C). It is active in vitro and in vivo (*Drosophila* model). It exhibited an inhibitory effect against various fungi apart from *Rhizopus* and is non-toxic. Although the targets of haemofungin were highly similar to the corresponding human protein, the authors suggest that this can be overcome, as the azoles currently in use as antifungals share 40% identity with a human protein [126].

#### 8.1.5. PC1244

PC1244 is a broad-spectrum antifungal active against various species of fungi, including *Mucorales* like *Rhizopus oryzae*, *Rhizomucor pusillus*, *Mucor circinelloides* and *Lichtheimia corymbifera*. It was found to have good activity in vitro against these fungi, where it demonstrated lower MICs compared to voriconazole and posaconazole. Additionally, it also shows rapid cellular permeation and persistence of action. The latter was observed when administered before inoculation in *Aspergillus fumigatus*, suggesting that it can be used for prophylaxis. It is proposed to act by inhibiting cell wall synthesis through inhibition of fungal sterol 14α-demethylase (CYP51A1) (Figure 3B). This study majorly focused on *A. fumigatus*. Further studies on *Mucorales* are required [127].

#### 8.1.6. EGFR Inhibitors

The host epidermal growth factor receptor (EGFR) is phosphorylated, activated, and colocalized with Mucorales fungi during infection. EGFR activation is critical for fungal invasion. As a result, network analysis identified EGFR as a potential drug target. Gefitinib (a drug) and Cetuximab (an antibody) are inhibitors of EGFR which were associated with lowered ability to invade fungi and more prolonged survival in mice with pulmonary mucormycosis. The response of EGFR to fungal infections is also reduced by gefitinib treatment [128].

### 8.2. Potential adjunct Drugs for Treatment of CAM

Various drugs have exhibited different interactions with existing medications to treat mucormycosis and therefore could potentially be used as combination therapies for CAM. These drugs and their activities have been described in detail. 

#### 8.2.1. Colistin

A study conducted by Ben-Ami et al., 2010 found that colistin had modest activity against mucormycosis [129]. It was demonstrated to act by affecting the cytoplasmic membrane by bleb formation adjacent to it and vacuolar membranes resulting in increased size and number of vacuoles. This collectively led to leakage of intracellular material, which is responsible for the fungicidal effect of colistin. When colistimethate was used in a murine model of pulmonary mucormycosis, the intranasal route (prophylaxis) was found to significantly impact the survival of mice compared to the intraperitoneal route (treatment), due to the possibility of attaining fungicidal concentrations in the lungs. However, colistin therapy alone was found to lead to regrowth, which was suppressed by using concentrations of Amphotericin B lower than the MIC. Hence, the authors proposed colistin as adjunctive therapy for mucormycosis. 

#### 8.2.2. HDAC Inhibitors

Pfaller et al., 2009 studied the effects of MGCD290, a Hos2 fungal histone deacetylase (HDAC) inhibitor, as monotherapy and in combination with triazoles [130]. Monotherapy had modest MICs, while synergistic activity was observed against most *Mucor* and *Rhizopus* fungi. Combination therapy was associated with synergy even in azoles to which these fungi are innately resistant (such as fluconazole). These effects are due to the suppression of Hos2 transcriptional complexes associated with resistance toward azoles.

#### 8.2.3. Miltefosine

Miltefosine, a membrane phosphatidylcholine analogue, was tested for activity against fungal pathogens as a monotherapy and in combination with voriconazole or posaconazole. The monotherapy exhibited high MICs, but in vitro synergy was observed with both azoles, as demonstrated by lowered MICs. Although it is known that Miltefosine targets fungal phospholipase B1 enzymes, the mechanism of synergy is unknown. Further in vivo studies are required [131].

#### 8.2.4. Statins

Lovastatin was found to be active against mammalian and fungal cells by generating apoptosis-like responses. In mouse models, it was found to act by inhibiting prenylation of signaling molecules such as Ras. In fungi, it led to morphology that resembled apoptotic cells, DNA degradation and loss of cell viability. However, it was ineffective in the spherical stage of fungal growth, possibly due to differences in metabolism from polarized growth [132]. It was found to improve the activity of voriconazole against *Rhizopus* and *Mucor* spp. in vitro. Synergy was observed with voriconazole against mucormycosis-infected models of *Drosophila*. However, studying the pharmacodynamics and pharmacokinetics of orally absorbed drugs is complex in *Drosophila* [133]. 

A study by Naeimi Eshkaleti et al., 2019 demonstrated that the combinations of Atorvastatin (synthetic statin) and Lovastatin (natural statin) with Amphotericin B led to a reduction of Amphotericin B MICs against *R. oryzae* [134]. Atorvastatin was found to cause a greater decrease of Amphotericin B MICs than Lovastatin. Statins and Amphotericin B are generally effective at higher concentrations, but these higher concentrations are also toxic to humans. A Statin-Amphotericin B combination reduces the harmful effects of both, improving activity.

#### 8.2.5. Rifampin

In combination with Amphotericin B, Rifampin demonstrated synergy against *Rhizopus* species in vitro. No significant effect was observed with Rifampin alone. This synergy was also observed in a patient with *Rhizopus* pneumonia. It is proposed to act by increasing cell permeability to Rifampin due to Amphotericin B binding with ergosterol. Rifampin entry results in DNA-dependent RNA polymerases inhibition, inhibiting fungal growth [135]. 

#### 8.2.6. Terbinafine

Terbinafine is an antifungal that inhibits fungal sterol synthesis, thus affecting ergosterol synthesis and cell wall synthesis. Terbinafine exhibited synergistic and additive effects against *Rhizopus*, *Rhizomucor* and *Mucor* species combined with amphotericin B and voriconazole [135]. The efficacy of terbinafine in animal models was poor [69]. 

#### 8.2.7. Quinolones

Quinolones are a class of bactericidal drugs that inhibit bacterial DNA replication by interfering with topoisomerase activity. Sugar and Liu, 2000 tested the effect of the Quinolone-Amphotericin B combination on pulmonary mucormycosis in a mouse model. The combination of fluconazole and trovafloxacin (a quinolone) was found to have improved median survival time (MST) compared to control and fluconazole monotherapy. Varieties of Amphotericin B-trovafloxacin and Amphotericin B-trovafloxacin-fluconazole were associated with longer MST than all other treatments (control, monotherapies, fluconazole-trovafloxacin combination therapy). Still, there was no significant difference in MSTs between these two treatments. Similar MST was also observed when the mice were administered fluconazole-ciprofloxacin treatment [136].

### 8.3. Immunomodulating Strategies

#### 8.3.1. Anti-CotH3 Antibodies

Anti-CotH3 binds to the receptor GRP78 and facilitates invasion. A predicted highly immunogenic and conserved domain present in the GRP78 binding domain of CotH3 was targeted using polyclonal antibodies. This was found to prevent invasion, angioinvasion and dissemination to the brain in DKA and neutropenic mice. It acts by multiple mechanisms, including increased phagocytic recruitment, higher phagolysosome acidification and increased ROS (Reactive Oxygen Species) production. Opsonophagocytosis helps in reducing the fungal burden. It might have a role in improving the fungicidal role of macrophages, further favouring its use in neutropenic patients [137].

#### 8.3.2. Anti-GRP78 Antibodies

Liu et al., 2010 demonstrated that blocking GRP78 using antibodies effectively prevented infection in mice with DKA [19]. Mucormycosis is an endothelial receptor critical for mucormycosis invasion. This method was not found to be effective in *Candida* or *Aspergillus*. This suggested the relevance of blocking GRP78 to treat mucormycosis. 

#### 8.3.3. Cytokine Administration

This includes interferon-γ (IFN-γ), granulocyte-colony stimulating factor (G-CSF), granulocyte macrophage-colony-stimulating factor and macrophage-colony stimulating factor (M-CSF). G-CSF and IFN-γ, in combination with GM-CSF, favour immune response by polymorphonuclear leukocytes (PMNs) and M-CSF promote the destructive activity of monocytes and macrophages. These are active against various invasive fungi in vitro and humans. Some combinations of cytokines act synergistically. IFN-γ is active against the broadest range of organisms [138]. IFN-γ, in variety with Nivolumab, has also helped reverse the effects of mucormycosis infection, which was unresponsive to existing therapy. G-CSF and GM-CSF have not been associated with reduced mortality but have suggested promoting shortened duration of neutropenia, lower antibiotic usage, and faster recovery. M-CSF has not been FDA-approved for administration to patients [139].

### 8.4. Other Therapies

#### 8.4.1. Photodynamic Therapy

Antimicrobial photodynamic therapy (aPDT) involves using a photosensitizer (PS), which sensitizes pathogenic fungi to the wavelength of light produced by an LED, resulting in a phototoxic reaction that produces reactive oxygen species, killing the fungi. This has been found to be useful for many pathogenic fungi, including *Rhizopus*. Pre-treatment with LED and methylene blue was observed to lower the MICs of existing antifungals used for mucormycosis treatment, such as itraconazole, posaconazole and amphotericin B. It is proposed as an alternative or adjunctive to surgical debridement owing to its high tissue transmission, localization to tissues with PS accumulation, non-invasiveness, low cost and convenience. Additionally, it can lower antifungal dosage and side effects, thus increasing patient compliance [140].

#### 8.4.2. Hyperthermia

Shirazi et al., 2013 conducted an in vitro study on the effects of hyperthermia on the activity of CNIs (tacrolimus) and triazoles (itraconazole and posaconazole) against *R. oryzae* [141]. It was observed that these drugs exhibited increased activity and lower MICs at higher temperatures in a dose-dependent manner. Higher temperatures were found to favour more elevated levels of ROS accumulation, leading to metacaspase activation and apoptosis. Hyperthermia was proposed as a therapy for mucormycosis, alone or combined with triazoles and tacrolimus. The authors suggest that local thermal delivery is a potential application of this finding. Further in vivo studies are required.

## 9. Insights from In Silico Studies 

A study by Jain et al., 2013 identified six potential targets based on sequence differences in humans. Out of these, three were shortlisted due to the presence of just one copy [142]. These are riboflavin synthase, riboflavin biosynthesis protein RibD domain-containing protein, and 3,4-dihydroxy-2-butanone 4-phosphate synthase. All these genes belong to the riboflavin synthesis pathway, which is essential in microorganisms and absent in humans. Studies are required to determine whether the organism can take up riboflavin from the host. Β-glucan synthase is involved in glucan synthesis, contributing to cell wall synthesis. A study by Sharma and Kaur identified 1–8 cineole, a bioactive compound from eucalyptus oil, as an inhibitor of this target using in silico methods [143]. They obtained a high-affinity docking score when the combination docked with the C-terminal end, responsible for catalysis. Further, they obtained good levels of pharmacokinetic and drug-likeness properties using online tools. 

## 10. Conclusions

This review discusses the risk factors and diagnosis associated with mucormycosis. Some possible links between COVID-19 and mucormycosis are also explored. Although only a few treatments are currently recommended to manage mucormycosis, other treatments must be explored due to the development of resistance to mucormycosis. Several therapies have been tested at various levels and have proved successful in treating mucormycosis. These treatments require further evaluation for administration to humans and treatment of CAM.

## Data Availability

Data sharing does not apply to this article.
